# Relationships Among Belief in God, Well-Being, and Social Capital in the 2020 European and World Values Surveys: Distinguishing Interpersonal and Ideological Prosociality

**DOI:** 10.1007/s10943-021-01411-6

**Published:** 2021-09-13

**Authors:** John B. Nezlek

**Affiliations:** 1 Institute of Psychology, SWPS University of Social Sciences and Humanities, Warsaw, Poland; 2 College of William & Mary, Box 8795, Williamsburg, 23185 USA

**Keywords:** Belief in God, Religiosity, Well-being, Prosocial, World Values Survey, European Values Survey

## Abstract

Analyses of the 2020 combined European and World Values Surveys (124,958 respondents from 77 countries) found that people who believed in God tended to be happier, more satisfied with lives, and healthier than non-believers. Believers trusted people close to them (e.g., neighbors) more than non-believers, although non-believers tended to trust people in general and trust people from other countries more than believers. Non-believers tended to be more ideologically prosocial than non-believers (e.g., belonging to an environmental organization, advocating freedom of speech vs. control). Such differences were stronger in countries in which there were more vs fewer believers. Moreover, these differences remained after controlling for individual differences in sex, age, education, income, and left–right political orientation.

## Introduction

A considerable body of research indicates that well-being is positively related to religiosity, with both constructs being defined in various ways (Koenig, 2012; Newman & Graham, 2018). Moreover, such relationships tend to occur in various cultures in which people follow a variety of religious faiths (Graham & Crown, 2014; Kim-Prieto & Miller, 2018; Tay et al., 2014). The positive effects of religiosity may also extend to increased longevity/decreased mortality, as suggested by a meta-analysis conducted by McCullough et al. (2000).

The present study was intended to complement existing research by examining relationships between belief in God (as a measure of religiosity) and social capital and well-being in a sample of approximately 125,000 people from 77 countries (a combination of the most recent World and European Values Surveys). The present study contributes to our understanding of these relationships in two important ways: (1) considering social capital as an indicator of well-being, and (2) proposing that relationships between religiosity and well-being and social capital can be explained by distinguishing interpersonal and ideological prosociality, a distinction described below.

### How to Define Well-Being?

Studies of relationships between well-being and religiosity have examined individually situated measures of well-being such as depression, happiness, affect, and so forth (Newman & Graham, 2018). Although valuable, research on relationships between well-being and religiosity needs to be expanded to include measures of well-being that represent people’s social integration or socially situated well-being. One such possibility consists of measures that are often referred to as social capital (e.g., Helliwell & Putnam, 2004).

Social capital includes trust in others, which can vary across different groups (e.g., family, friends, neighbors, etc.), and it includes social, political, and civic participation, among other aspects of social/community life (Putnam, 2000). Strictly speaking, social capital is not usually considered a measure of well-being, which is usually defined in terms of individual states (e.g., life satisfaction and happiness); rather, social capital is treated as something that enables or promotes well-being. Nevertheless, the relationships between social capital and well-being are so pervasive and well-documented that social capital can be treated as a proxy measure or indicator of well-being. Moreover, relationships between religiosity and social capital can inform our understanding of relationships between religiosity and well-being per se.

The present study follows a suggestion made by Helliwell and Putnam (2004, p. 1437), a suggestion that I do not believe has been followed in terms of relationships between religiosity and social capital: “Similarly, we need to distinguish among different types of social capital, like the difference between ‘bonding’ social capital—these are links among people who are similar in ethnicity, age, social class, etc.—and ‘bridging’ social capital, which are links that cut across various lines of social cleavage.” As explained below, these two types of social capital can be seen as manifestations of interpersonal prosociality (bonding social capital) and ideological prosociality (bridging social capital). Moreover, the present study goes beyond links between individuals and considers social capital (or ideological prosociality) in terms of attitudes and beliefs about society broadly defined.

### The Distinction Between Interpersonal and Ideological Prosociality

Generally speaking, research has found positive relationships between religiosity and prosociality (e.g., Myers, 2012). Moreover, such relationships seem to be a reflection of the underlying values of religious belief systems. For example, as noted by Stavrova and Siegers (2014, p. 315), “All world religions contain ethical principles that prescribe prosocial and other-regarding behavior.” Similarly, in their review, Miller et al. (2012) emphasized the centrality of altruism to various religious beliefs. Although not all prosocial acts may be altruistic, altruistic acts are clearly prosocial.

Prosociality is typically defined in terms of behaviors such as helping people or trust in others; however, prosociality can also be conceptualized in terms of the attitudes and beliefs people hold. For example, an individual may favor social policies that emphasize collective well-being such as universal access to health care. Such advocacy is intended to help others, but such advocacy also represents a qualitatively different phenomenon from helping someone cross a street. I believe that these two types of prosociality reflect distinct domains of prosociality, which are labeled as interpersonal and ideological prosociality. The interpersonal domain includes how people think about and behave toward others in their personal lives (helping, providing social support, etc.), whereas the ideological domain includes attitudes and behaviors relevant to social policies, collective action, and so forth.

The following illustrate the distinction between the two types of prosociality. Someone may volunteer to work at a charitable organization, and she may be responsible for organizing a fund-raising drive. Is her work on this drive prosocial? Absolutely. Is it interpersonally prosocial? It is difficult to see how it is. Similarly, someone may participate in a rally or sign a petition supporting women’s rights. Are these prosocial acts? Certainly. Are they interpersonally prosocial? It is difficult to see how they are—neither behavior is directed toward a specific person or persons.

Unfortunately, the WVS-EVS combined survey did not measure directly either interpersonal or ideological prosociality (most cross-national surveys do not). Nevertheless, the survey contains data that can be used to address this topic, albeit indirectly, e.g., bonding and bridging social capital. The WVS-EVS also measured attitudes that do not concern social capital but do concern ideological prosociality, e.g., the nature of personal goals, priorities for society, materialism.

These data provided a basis for drawing inferences about relationships between religiosity and the motives and values that might underlie different types of social capital and personal goals, priorities, and materialism. Such inferences are important because measures of social capital and measures of goals, priorities, and materialism do not measure why people possess certain attitudes and beliefs. They simply measure the extent to which people believe something to be true or the extent to which they endorse or advocate a position. The issue of drawing conclusions about prosociality from the data available in the WVS-EVS is addressed in the discussion.

### Representing Religious Beliefs with a Common Metric for Different Beliefs and Cultures

Examining relationships between religious beliefs and social capital and well-being in different cultures requires measures for all constructs that are valid across the range of cultures in a study, and the measures that were collected in the World and European Values Surveys provided a reasonable basis to examine such relationships. Deciding to focus in the present paper on belief in God was made with the recognition that the WVS is global in scope. The combined survey that was analyzed for this paper has over 125,000 respondents living in 79 countries from all six inhabited continents.

To avoid the difficulties inherent in using multiple categories representing various faiths, all or many of which would not exist in many countries, and to avoid the problems with the ambiguity inherent in many measures of religious behavior, the analyses in this paper focused on whether people believed in God. Belief in God is a foundation of religious faith, and various measures of religiosity are manifestations of this fundamental belief. Admittedly, such a distinction does not take potentially important differences among faiths into account, e.g., differences among Christians, Jews, and Muslims in their beliefs about the God of Abraham, but given the need to find a measure that would be relevant for measuring beliefs among the followers of the various religions of the world, belief in God per se seemed like a good option, or at least a good place to start.

In addition to questions about well-being, the WVS-EVS asks a series of questions about social capital. These include questions about how much respondents trust groups of people (e.g., family members, people in their neighborhoods), and there are also questions about how important family and friends are in their lives (bonding social capital). There were also items that measured bridging social capital and ideological prosociality. These included memberships in organizations (charities, environmental/conservation), prioritizing certain policies over other policies (e.g., free speech over national defense), and civic involvement (e.g., signing petitions).

### General Expectations: Relationships Between Belief in God and Well-Being

Previous research has consistently found that religiosity is positively related to well-being, and so I expected that people who believed in God would report greater well-being than people who did not believe in God. Although such relationships do not directly address relationships between religiosity and social capital and prosociality, they add to the body of research on the topic of relationships between well-being and religiosity.

As discussed previously, most religious belief systems encourage followers to act prosocially, e.g., to be kind and helpful to others. This suggests that those who believe in God should be more prosocial than non-believers, no matter how prosociality is defined (e.g., as bonding or bridging social capital). Nevertheless, there are reasons to believe that relationships between religiosity and prosociality might vary as a function of whether interpersonal or ideological prosociality is being examined.

For example, Preston et al. (2010) discuss research, suggesting that greater religiosity is associated with reduced prosociality toward outgroup members, particularly when people believe that outgroup members threaten core values. Such concerns about ingroup protection may be particularly relevant to the present construct of ideological prosociality, which is defined in terms of prosociality toward people who are not necessarily part of one’s ingroup. Given this, I expected that ideological prosociality would be negatively related to belief in God.

In terms of interpersonal prosociality as measured in the WVS-EVS, what Preston et al. described as the religious principle should be associated with increased prosociality for some groups (e.g., greater trust in family) and decreased prosociality for other groups (e.g., people from other countries). This distinction also parallels the distinction between bonding and bridging social capital.

### The Role of Context

The preceding discussion has concerned what could be called in statistical terms “main effects.” Ignoring other considerations, do people who believe in God enjoy greater well-being and do they act more prosocially than people who do not believe in God? Although informative, such comparisons can provide an incomplete picture. People’s beliefs in God exist within contexts, e.g., the societies in which they live. For example, believing in God when most others in your society also believe renders such beliefs normative, whereas believing in God when most others in your society do not believe makes believers members of a minority.

Research on how relationships between religiosity and well-being vary as a function of contextual effects has examined a wide variety of contextual effects. The present study examined the moderating (contextual) effect of the societal (country) level of belief in God. Do relationships between belief in God and well-being and between belief in God and prosociality vary as a function of how normative belief in God is in a country?

I chose this measure for two reasons. First, it is a direct extension of the individual-level variable of belief in God. Second, previous research has found that individual-level relationships between religiosity and well-being vary as a function of how normative religious belief is in a country (e.g.,, Stavrova et al., 2013), and the percent of believers is a clear measure of norms.

As discussed by Kim-Prieto and Miller (2018), the upshot of research on the moderating effects of national levels of religiosity is that individual-level relationships between religiosity and well-being tend to be stronger in countries in which religiosity is more normative than they are in countries in which religiosity is less normative. For the present study, I expected that relationships between belief in God and well-being and between belief in God and social capital would be stronger in countries in which a greater percent of people believed in God than in countries in which a lower percent believed in God.

### Study Objectives

1. To examine relationships between belief in God and well-being and social, and to determine if such relationships are consistent with or support a distinction between interpersonal and ideological prosociality.
2. To determine if the relationships found between belief in God and well-being and social capital vary across countries as a function of how normative belief in God is in a country.

## Methods

### Sample

The data analyzed in the present paper were taken from a dataset created by joining overlapping World and European Value Surveys. This merging was done by a committee composed of representatives of the WVS and EVS. The World Values Survey (WVS) is the preeminent survey of its kind. Stratified random samples are taken in each country that participates, and the same questions are administered in each country. These questions are formulated by a committee of experts from across the world representing numerous disciplines. The WVS and EVs are conducted approximately every ten years. The European Values Survey (EVS) is similar to the WVS in terms of rigor, but it is limited to countries in Europe.

The specific surveys that were combined were Wave 7 of the WVS, conducted between 2017 and 2020, and Wave 5 of the EVS, also conducted between 2017 and 2020. A description of the sampling and methods is available from either of the organizing committees (EVS/WVS, 2020). The combined sample contains data from 127,358 respondents in 79 countries. In two countries, Iran and Egypt, respondents were not asked directly if they believed in God, and so the present analyses examined a sample of 124,958 respondents from 77 countries. Note that respondents were not required to answer any question, and for this and other reasons, the number of observations varied slightly across the analyses of different measures. Also note that the WVS and EVS are both cross-sectional.

### Measures

#### Belief in God and Well-Being

The questions in the EVS/WVS cover a variety of topics, some of which are directly relevant to the topics at hand. First, and most important, there was a question about belief in God: “Do you believe in God?” with a yes/no response option. Two questions measured psychological well-being, one about happiness, “Taking all things together, would you say you are… 1 = very happy, 4 = not at all happy,” and another about life satisfaction, “All things considered, how satisfied are you with your life as a whole these days… 1 = dissatisfied, 10 = satisfied.” There was also a self-rating of state of health, “All in all, how would you describe your state of health these days? Would you say it is…” 1 = very good, 5 = very poor.”

Prior to analysis, responses were scored as follows. As an outcome, belief in God was coded 0 = no, 1 = yes. As explained below, when belief in God was a predictor, responses to this item were coded as − 1 = no and 1 = yes. Responses to the happiness and health items were reverse scored so that higher scores indicated greater happiness and better health.

#### Interpersonal Prosociality and Bonding/Bridging Social Capital

Interpersonal prosociality refers to the positive attitudes, dispositions, and behaviors people have or exhibit to other people. The following measures were taken from the EVS/WVS as indicators of interpersonal prosociality. First, respondents indicated how important family and friends were in their lives. Responses were made using a 4-point scale (1 = very important, 4 = not at all important). Second, respondents indicated how much they trusted members of their families, people in their neighborhoods, people they know personally, people they met for the first time, people of another religion, and people of another nationality. Responses were made using a 4-point scale (1 = trust completely, 4 = do not trust at all). Prior to analysis, responses to these items were reverse scored so that higher scores indicated greater importance and more trust. Finally, respondents indicated how much they trusted people in general. Responses were coded 0 = you can’t be too careful, 1 = most people can be trusted.

Although these items all refer to the interpersonal domain, they differ in terms of whether they refer to bonding or bridging social capital. Questions about family and friends, and people in one’s neighborhood concern bonding social capital, whereas questions about people in general and people from other countries concern bridging social capital.

#### Ideological Prosociality and Civic Involvement

Ideological prosociality refers to the attitudes and values people hold regarding social policies and how society should be organized and governed, and it includes behaviors that are intended to benefit others collectively. Some of these attitudes, values, and behaviors overlap with what social capital researchers refer to as civic involvement. The following were taken from the EVS/WVS as indictors of ideological prosociality: Being a member of a charitable organization and being a member of an organization dedicated to conservation, the environment, ecology, or animal rights. Both were coded as 0 = no, 1 = yes. Political actions: signing a petition, joining in boycotts, attending lawful/peaceful demonstrations, and joining unofficial strikes. All were coded as 0 = would never do, 1 = might do/have done.

In a series of questions, respondents indicated what their priorities were. One question asked respondents to select economic growth and creating jobs vs. protecting the environment, and these were coded as 0 and 1, respectively. Respondents also indicated their aims for the future of their country. The first question asked them to pick their top choice out of four options: (1) A high level of economic growth, (2) Making sure this country has strong defense forces, (3) Seeing that people have more say about how things are done at their jobs and in their communities, or (4) Trying to make our cities and countryside more beautiful. The next question asked for their second choice from the same four options. For both questions, selecting item 1 or 2 was coded as 0, and selecting item 3 or 4 was coded as 1.

Finally, I analyzed what is called the post-materialism index, originally formulated by Inglehart (1977). The index reflects the extent to which individuals place greater importance on non-material goals such as freedom of speech, gender equality, self-expression, and environmentalism than they do on material goals. More details about how this measure was calculated can be found on the website for the survey (EVS/WVS, 2020). Although the index is calculated using some of the items that have been mentioned previously (e.g., priorities for the future), given its widespread use, it was analyzed for the present paper. Individuals were classified as either materialist, mixed, or post-materialist.

## Results

### Overview of Analyses

The data were conceptualized as a multilevel data structure in which persons were treated as nested within countries. Accordingly, the data were analyzed with a series of multilevel models (MLM) using the program HLM (Raudenbush et al., 2011). In essence, in these analyses, a set of regression coefficients was estimated for each country and mean coefficients were estimated. These analyses also provided a basis for examining between-country differences in individual-level relationships. For example, do differences in well-being between believers and non-believers vary as a function of the overall level of belief in God in a country (a contextual effect)? A rationale for using MLM to analyze multi-national datasets such as the EVS/WVS and guidelines for conducting such analyses are presented in Nezlek (2010).

### Basic Model: Structure of Analyses and Descriptive Statistics

The basic model representing the nested structure of the data is presented below. In this model, y is an outcome, and there are *i* people nested within *j* countries. A mean (β_0j_) is estimated for each of j countries, and the overall mean is γ_00_. The within-country variance (how much do people vary around the mean for their country) is the variance of r_ij_, and the between-country variance (how much do the means of countries vary) is the variance of μ_0j_.

-
  Individual-level: y_ij_ = β_0j_ + r_ij_.
  Between-country: β_0j_ = γ_00_ + μ_0j_.

Although the logic of the analyses of categorical outcomes is the same as the logic for continuous outcomes, in MLM, a different estimation algorithm is used for non-continuous outcomes (e.g., yes /no responses) than for continuous outcomes, and variance estimates comparable to those for continuous outcomes are not estimated. In place of these variance estimates, 95% confidence intervals for the mean percent of these measures are presented. See Raudenbush and Bryk (2002, pp. 301–310) for a discussion. In all analyses, observations were weighted using the GWEIGHT measure provided by the EVS/WVS.

Summary statistics for the measures are presented in Tables 1, 2, and 3. These summary statistics include the mean (or mean percent), the within- and between-country variance estimates for continuous measures, and 95% confidence intervals for dichotomous outcomes. Given that belief in God is treated as a predictor, not as an outcome, summary statistics for this measure are not provided in the tables. Across all countries, 79% of 120,136 respondents indicated they believed in God, and the 95% confidence interval for this percent was 74–84%.

**Table 1:** Belief in God and well-being: descriptive statistics, differences as a function of belief in God, and the moderating effect of normative belief in God controlling for sex, age, income, education, and political orientation

Outcome	Mean	Variance estimates		Belief in God: Coefficients and estimated means				Country mean: Moderating effect and estimated difference			
		Within	Between	γ_10_	*t*-ratio	No	Yes	γ_11_	*t*-ratio	High	Low
Happiness	3.14	.439	.039	.049	7.78***	3.06	3.16	.022	3.85***	.144	.076
Life satisfaction	7.21	4.33	.474	.118	5.08***	7.02	7.26	.059	2.81**	.353	.176
Self-rated overall health	3.79	.769	.050	.028	4.12***	3.74	3.79	.016	2.35*	.089	.040

Happiness was measured using a 1–4 scale, life satisfaction was measured using a 1–10 scale, and self-rated health was measured using a 1–5 scale. Main effects from final models with moderator

***p < .001; **p < .01; *p < .05

**Table 2:** Interpersonal prosociality: descriptive statistics, differences as a function of belief in God, and the moderating effect of normative belief in God

Outcome	Mean	Variance estimates		Belief in God: Coefficients and estimated means				Country mean: Moderating effect		Belief effect country mean	
		Within	Between	γ_10_	*t*-ratio	No	Yes	γ_11_	*t*-ratio	High	Low
Importance of family	3.89	.135	.004	.034	9.18***	3.83	3.90	.004	< 1		
Importance of friends	3.32	.461	.042	.006	1.71^a^	3.32	3.33	.006	< 1		
Trust											
Family	3.77	.252	.018	.029	6.07***	3.71	3.77	.014	3.00***	.087	.045
Neighbors	2.85	.550	.062	.030	4.66***	2.81	2.86	.011	2.21*	.081	.048
People you know	2.98	.490	.093	.003	< 1						
People meet first time	2.04	.548	.093	− .014	2.02*	2.10	2.07	.015	2.29*	.060	.014
People other religions	2.38	.609	.100	.032	5.06***	2.38	2.45	− .010	1.73^a^	.038	.069
People other countries	2.32	.596	.137	− .017	1.94*	2.41	2.37	− .017	4.64***	.107	.002
General trust	22%	CI: 18–26%		− .039	2.34*	24.6%	22.9%	− .037	1.76^a^	1.8%	1.2%

Includes covariates. Importance and trust in specific types of people were measured using a 1–4 scale. The last measure was dichotomous. When moderator was significant, estimates of main effects are from analyses containing the moderator

***p < .001; **p < .01; *p < .05; ^a^*p* < .10

**Table 3:** Ideological prosociality: descriptive statistics, differences as a function of belief in God, and the moderating effect of normative belief in God

Outcome	Descriptive statistics		Belief in God: Coefficients and estimated means				Country mean: Moderating effect		Belief effect country mean	
	Mean (%)	CI (%)	γ_10_	*t*-ratio	No (%)	Yes (%)	γ_61_	*t*-ratio	High (%)	Low (%)
Priority: environment vs. economy	61	58–63	− .001	< 1						
Belong to environmental organization	7	6–9	− .089	3.20**	9.2	7.8	− .058	2.23*	1.9	.9
Belong to charitable organization	11	9–13	.032	1.21						
First choice aim for country	31	28–34	− .103	5.27***	35.1	30.5	− .055	3.16**	6.5	3.5
Second choice aim for country	37	34–39	− .043	2.48*	38.2	36.2	− .026	1.32		
Sign petition (yes/might)	62	56–68	− .019	< 1						
Join boycott (yes/might)	36	32–41	− .011	3.47**	46.2	40.8	− .054	1.73^a^	6.4	4.2
Attend lawful demonstrations (yes/might)	50	46–54	− .059	1.98*	61.2	58.4	− .059	1.93^a^	4.4	1.4
Join unofficial strikes (yes/might)	30	26–34	− .090	3.27**	37.4	33.3	− .042	1.37		
Materialist	26	23–29	.142	7.14***	18.9	24.5	.047	2.12*	6.9	4.3
Mixed materialist/post-materialist	60	58–61	.000	< 1						
Post-materialist	11	9–13	− .173	7.11***	14.6	10.8	− .050	2.18*	4.0	3.5

See note for Table 2 for *p* values, use of covariates, and estimates of main effects

### Controlling for Sociodemographic Differences and Political Orientation

To control estimates of relationships between belief in God and measures of well-being and manifestations of prosociality for individual differences in sociodemographic characteristics, the following covariates were included: (1) respondent gender, represented with a contrast-coded variable (sexcnt; 1 = women, − 1 = men), (2) age, (3) income measured in deciles, education (ISCED; UNESCO, 2011), and left–right orientation (0–10 scale, 0 = left and 10 = right).

Including these covariates reduced the sample size from 120,136 respondents who answered the belief in God question to approximately 80,000 for most analyses. Much of this reduction was because 32,267 participants did not answer the left–right orientation question. Although including this covariate meaningful reduced the sample size, left–right orientation was strongly related to belief in God (*p* < 0.0001), and so including this covariate strengthened the confidence in the results. Moreover, these missing responses were distributed relatively evenly across the countries, and the effects for belief in God from analyses that did not include left–right orientation were largely the same as the effects from analyses that included left–right orientation as covariate.

Summaries of the analyses that did not include left–right orientation as a covariate are available in the supplemental materials. The supplemental materials also include a summary of the number of participants in the analyses presented in the paper and analyses without left–right orientation as a covariate. These files are available via the OSF data repository (Nezlek, 2021a).

### Differences in Well-Being and Prosociality/Social Capital as a Function of Belief in God

To compare the well-being and prosociality of respondents who believed in God with those who did not, a contrast-coded variable representing belief in God, Belief Cnt, coded 1 = yes, − 1 = no, was added at the individual level to the basic model presented above. All the covariates except for the sex contrast variable were entered group-mean centered. The sex contrast variable was entered uncentered. See Enders and Tofighi (2007) for a discussion of centering. All predictors were modeled as randomly varying (Nezlek, 2010). The statistical significance of differences between believers and non-believers (i.e., was the mean coefficient representing the contrast of believers and non-believers different from 0) was tested at the between-country level by testing the statistical significance of the γ_10_ coefficient. In the interests of brevity, not all of the country-level equations are presented. The full model is below.

-
  Individual-level: y_ij_ = β_0j_ + β_1j_ * (BeliefCnt) + β_2j_ * (SexCnt) + β_3j_ * (Age) + β_4j_ * (Income) + β_5j_ * Education + β_6j_ * (Left–Right) + r_ij_.
  Between-country: β_0j_ = γ_00_ + μ_0j_(Intercept).
    β1j = γ10 + μ1 (Belief in God).
    (same for other predictors).
    β6j = γ50 + μ6j (Left–Right orientation).

Note that the intercepts in these analyses (γ_00_) were not necessarily the same as the intercepts from the unconditional model because these intercepts were adjusted for individual differences in the predictors. These intercepts are not presented in the tables, but they were used to calculate estimated values.

### Well-Being

The results of the analyses of well-being, including expected values for respondents who believed in God and those who did not, are summarized in Table 1. There were significant effects for belief in God in the analyses of happiness, satisfaction with life, and self-reported health. On average, individuals who believed in God were happier than those who did not, they were more satisfied with their lives, and they reported better physical health.

### Interpersonal Prosociality and Bonding/Bridging Social Capital

The results of the analyses of interpersonal prosociality, including expected values for respondents who believed in God and those who did not, are summarized in Table 2. On average, individuals who believed in God thought that family and friends were more important than those who did not believe in God, and believers trusted family members, neighbors, and members of other religions more than non-believers did. In contrast, respondents who believed in God trusted people they meet for the first time and people from other countries less than non-believers did, and believers’ general trust in people was lower than non-believers’ general trust in people.

Note that the differences in the relationships between belief in God and these outcomes correspond to the distinction between bonding and bridging social capital. Measures of bonding social capital were positively related to belief in God, whereas measures of bridging social capital were negatively related. Nevertheless, some of these differences, although statistically significant, were very small (i.e., importance of friends).

### Ideological Prosociality and Civic Involvement

The results of the analyses of ideological prosociality, including expected values for respondents who believed in God and those who did not, are summarized in Table 3. There were significant effects for belief in God for eight of the twelve measures: belonging to an organization concerning environmentalism, aims for the country, joining boycotts, attending lawful demonstrations, joining unofficial strikes, and being classified as materialist and as post-materialist. The results of these analyses indicated that people who believed in God were less ideologically prosocial and were less civically involved than those who do not believe in God.

#### Normative Religiosity as a Moderator of Individual-Level Relationships Between Belief in God and Well-Being and Prosociality

To examine how individual-level relationships between belief in God and well-being and prosociality varied as a function of country-level normative beliefs, the percent of respondents in a country that indicated they believed in God was entered at the between-country level as shown below. These analyses were limited to outcomes for which the individual-level belief in God effect was significant. The moderating effect was tested by the γ_11_ (Pct Believers) coefficient. If it was significantly different from 0 this indicated that there was moderation.

Individual-Level: y_ij_ = β_0j_ + β_1j_ (BeliefCnt) + r_ij_.
Between-country: β_0j_ = γ_00_ + γ_01_ (Pct Believers) + μ_0j_.
  β_1j_ = γ_10_ + γ_11_ (Pct Believers) + μ_1j_.

To ease the interpretation of the results, the percent of believers in a country was standardized prior to analysis (*M* = 79%, *SD* = 21), and so percent of believers was entered uncentered. In regression analyses, predicted values are usually estimated for units that are ± 1 *SD* from the mean. Given that the estimated percent of believers for a country that was + 1 SD on percent of believers was 100% (not a realistic reference point), predicted values for countries that were ± 0.5 *SD* from the mean (approximately 90% and 68% believers, respectively) were estimated. The estimated differences between believers and non-believers for countries high in belief in God (+ 0.5 SD) and for countries low in belief in God (− 0.5 SD) are presented in Tables 1, 2, and 3.

The results of the analyses were largely consistent with the expectation that differences between believers and non-believers would be larger in countries in which belief in God was more normative than in countries in which belief in God was less normative. As can be seen in Table 1, this was the case for happiness, life satisfaction, and self-rated health. The difference between believers and non-believers was twice as large in high-belief countries than it was in low-belief countries.

For interpersonal prosociality (Table 2), the moderating effect was significant for all six outcomes for which the belief in God effect was significant. The difference between believers and non-believers was larger in countries in which there were more believers than in countries in which there were fewer believers, except for trust in people from other religions, for which the difference was in the opposite direction.

The analyses of ideological prosociality produced similar results, which are summarized in Table 3. The moderating effect was significant for four of the eight outcomes for which the belief in God effect was significant, and it was significant at *p* < 0.10 for two measures. In all of these cases, the difference between believers and non-believers was larger in countries in which the percent of believers was higher than it was in countries in which the percent of believers was lower.

## Discussion

As expected, individuals who believed in God reported being happier and being more satisfied with life than individuals who did not believe in God. Moreover, this difference was greater in countries in which more people (percent) believed in God than in countries in which fewer people believed in God. Such differences are consistent with much of the previous research on this topic. As discussed by Kim-Prieto and Miller (2018), a variety of explanations have been offered for such relationships; unfortunately, the combined WVS/EVS dataset does not contain the information necessary to examine such possibilities.

Nevertheless, it is valuable to demonstrate that such relationships exist in a time when many have suggested that belief in God has lost or is losing its relevance. For example, Inglehart (2020) provides a strong argument for an international decline in religiosity, but does not discuss changes in belief in God per se; he focuses primarily on measures of religiosity such as the importance of religion. Moreover, Inglehart focuses on declines in religiosity, not on changes in the relationships between religiosity and well-being. A decline in the strength of religious beliefs does not necessarily entail a change in the strength of the relationships between religiosity and well-being. Even if religiosity has declined, it appears that belief in God, which is related to, but is not the same as religiosity, remains an important predictor of well-being. It is possible that relationships between belief in God and religiosity are decreasing over time, but the present data cannot address such questions.

### Context Effects

For many of the measures for which the overall difference between believers and non-believers was significant, these differences were larger in countries in which there were more believers than in countries in which there were fewer believers. Various explanations have been given for such relationships (Kim-Prieto & Miller, 2018), and the present study could not address all of these explanations. Nevertheless, the context effects found in the present study could reflect the salience of believers’ majority status.

Of the 77 countries included in the analyses, only ten had a mean percent of believers less than 50%, and the percentages for five of these ten were between 45 and 49%. In only one country (China, 17%) was the percent of believers lower than 38%. So, for the most part, believers were members of the majority or were members of sizable minorities. Assuming that the percent of believers in a country is reflected in various ways (e.g., civil and cultural observance of religious holidays), the salience of belief in God will be greater when there are more believers in a society than when there are fewer. In turn, greater salience should result in larger effects (positive and negative) for belief in God. Examining such possibilities will require further study with measures that are designed to examine them.

### Inferring Prosociality from Measures of Social Capital and Measures of Attitudes and Beliefs

The most important limitation of the present study was that there were no direct measures of prosociality. Unfortunately, prosociality (of any kind) is rarely, if ever, measured directly in large-scale cross-national surveys such as the EVS and WVS. Nevertheless, I believe that the present results support the existence of interpersonal and ideological prosociality. The logic behind this is that belief in God was positively related to measures of what has been defined as interpersonal prosociality, whereas it was negatively related to measures of what has been defined as ideological prosociality. Admittedly, there may be other distinctions that could account for this pattern of results, but the distinction between these two types of prosociality is able to account for the present results.

Moreover, the results of preliminary analyses of the European Social Survey (ESS8) from 2016 (Nezlek, 2021b) support the contention that what have been described as manifestations of ideological and interpersonal prosociality represent different values. The ESS8 measured a set of values proposed by Schwartz (2001), and two of these values were Universalism and Benevolence, which correspond to ideological and interpersonal prosociality. Nezlek (2021b) found that ideological prosociality (Universalism) was positively related to support for ideological prosocial policies (or bridging social capital) such as environmentalism and income equality, whereas with one exception, interpersonal prosociality was not related to such support. In contrast, interpersonal prosociality (Benevolence) was positively related to measures of bonding social capital such as the ability to discuss intimate matters with others, whereas ideological prosociality was not related to such measures. Unfortunately, belief in God was not measured in the ESS8, so differences between believers and non-believers could not be examined.

### Implications for Understanding Relationships Between Religion and Health

The present study replicated previous research about relationships between religiosity and well-being. Those who believed in God reported greater life satisfaction, happiness, and better health than those who did not believe. There are numerous explanations for such relationships, but most of these explanations rely upon intervening variables such as meaning in life, coping with stress, social support, and emotions (Koenig, 2012; Newman & Graham, 2018), that were not measured in the WVS-EVS. Nevertheless, the scale of the present study and the number of covariates that were included in the analyses provide additional and strong support for these empirical relationships.

Perhaps the most important contribution of the present study consists of the results concerning relationships between belief in God and social capital. Understanding such relationships is important because social capital is a precursor to well-being and determines (or reflects) the potential a social environment has to affect well-being. Over time, greater social capital is associated with greater involvement in communities, which in turn is associated with enhanced well-being (Helliwell & Putnam, 2004), and conversely, decreases in social capital are associated with decreases in well-being.

To the extent that belief in God is positively related to social capital, belief in God will be positively related to well-being or has the potential to facilitate well-being. In terms of bonding social capital (e.g., quality of personal relationships), which has been interpreted as a manifestation of interpersonal prosociality, belief in God seems to predispose people to have greater bonding social capital. In contrast, in terms of what has been interpreted as manifestations of ideological prosociality, bridging social capital (e.g., relationships with strangers) and measures of civic involvement (joining boycotts, attending demonstrations, etc.), belief in God seems to predispose people to have less social capital.

The possible upshot of these findings is that the belief in God may lead believers to separate themselves from non-believers. Believers trust family and friends more than non-believers, but they trust strangers less than non-believers. This may be an indication that believers’ social networks consist primarily of other believers. Moreover, believers are less likely than non-believers to participate in demonstrations, boycotts, and strikes. Believers may have a more circumscribed view of the world than non-believers, something that is suggested by believers’ greater endorsement of materialist values and weaker endorsement of post-materialist values compared to non-believers. It is important to note that these relationships existed after controlling for sex, age, education, income, and political orientation.

The foregoing argument has placed the responsibility for a possible lack of cross-group social integration with believers. Such an argument is consistent with the presumed bases for the outcomes that were examined, ideological and interpersonal prosociality. However logical this argument may be, the proposed model requires empirical support.

Social capital can be conceptualized in terms of the interpersonal closeness that is part of the nature of social integration. Believers may be better integrated than non-believers in terms of closer social integration. In contrast, non-believers may be better integrated in terms of their broader societies. How the influences on well-being of these two sources will change as religious identification, and possibly, belief in God, change, remains to be seen.

### Effect Sizes

Although calculating effect sizes in MLM is not straightforward (e.g., Nezlek, 2011), when they could be estimated, the effect sizes of the significant effects were at best, modest, less than 5% of the variance, and often lower than that. Such effect sizes are similar to the effect sizes found in previous research using large-scale surveys. Nevertheless, such small effects are to be expected when analyzing outcomes such as well-being that represent the total sum of a myriad of influences.

Moreover, it needs to be kept in mind that some of these relationships were between what is probably a relatively stable measure (belief in God, yes/no) and measures that might fluctuate over time (e.g., happiness) but were measured at a single point in time. It is possible, perhaps even likely, that relationships between belief in God and such measures would be stronger if outcomes were measured multiple times, e.g., every day for two weeks. Multiple measures are more reliable than single assessments, and such multiple measurements reduce the impact that immediate circumstances (e.g., having a good or a bad day) have on single assessments (Newman et al., 2020; Nezlek, 2012).

Regardless, the substantive significance of the present effects needs to be considered in light of the number of people to which the effects refer. For example, belief in God was associated with a 1.5% difference in the likelihood that people were members of environmental/conservation organizations. Such a difference is larger than the margin of victory in many contemporary elections. In terms of people’s goals, differences between believers and non-believers in advocating prosocial goals were 4% and 8%, which are also meaningful differences.

The point here is not to defend small effect sizes per se. Rather, the point is to recognize that small effects may be worth considering when they have consequences for a large number of people. Belief in God is just one of the many factors that influence well-being, social capital, and prosociality, but the majority of people in the world believe in God, and so this influence is manifested across the world.

## Limitations and Future Directions

An important limitation of archival research is that a study may not include the best measures of the constructs of interest, and this was the case with the EVS/WVS combined dataset as a study of prosociality. Although some of the measures were perfectly appropriate (e.g., satisfaction with life, trusting other people), others were less than ideal. Nevertheless, on balance, the nature of the sample (stratified random samples from 77 countries) may have offset the shortcomings due to any lack of correspondence between the items and the constructs of interest.

The overarching assumption of the present analyses was that belief in God leads to certain outcomes rather than the reverse. Although belief in God may be a stable individual difference, it may not be. Moreover, the extent to which people’s beliefs in God change calls into question the causal link from belief to the other measures that was assumed in the present study. The present study was cross-sectional, and causal links between belief in God and well-being can be examined only through longitudinal studies designed to address such questions.

The present study demonstrated that belief in God per se is related to well-being, and the results are also consistent with a model of prosociality that distinguishes interpersonal and ideological prosociality. Nevertheless, establishing empirical relationships is a necessary, but not sufficient, condition for understanding how and why constructs are related. Clearly, more research is needed to determine the validity of the distinction between interpersonal and ideological prosociality. Regardless, future research should take into account the fact that despite declines in religiosity as traditionally defined, the vast majority of the world’s population still believes that God exists, and such beliefs are associated with meaningful outcomes.

## Data Availability

All data are available from the following EVS/WVS website: 10.14281/18241.2.

## References

[CR1] Enders CK, Tofighi D (2007). Centering predictor variables in cross-sectional multilevel models: A new look at an old issue. Psychological Methods.

[CR2] EVS/WVS. (2020). *European Values Study and World Values Survey: Joint EVS/WVS 2017-2021 Dataset (Joint EVS/WVS). Dataset Version 1.0.0*. 10.14281/18241.2

[CR3] Graham C, Crown S (2014). Religion and wellbeing around the world: Social purpose, social time, or social insurance?. International Journal of Wellbeing.

[CR4] Helliwell JF, Putnam RD (2004). The social context of well-being. Philosophical Transactions of the Royal Society of London. Series b: Biological Sciences.

[CR5] Inglehart R (1977). The silent revolution: Changing values and political styles among western publics.

[CR6] Inglehart RF (2020). Giving up on God: The global decline of religion. Foreign Affairs.

[CR7] Kim-Prieto C, Miller L, Diener E, Oishi S, Tay L (2018). Intersection of religion and subjective well-being. Handbook of well-being.

[CR8] Koenig HG (2012). Religion, spirituality, and health: The research and clinical implications. ISRN Psychiatry.

[CR9] McCullough ME, Hoyt WT, Larson DB, Koenig HG, Thoresen C (2000). Religious involvement and mortality: A meta-analytic review. Health Psychology.

[CR10] Miller LJ, Mullin ASJ, Barkin SH (2012). Religion, altruism, and prosocial behavior: Conceptual and empirical approaches.

[CR11] Myers DG (2012). Reflections on religious belief and prosociality: Comment on Galen (2012). Psychological Bulletin.

[CR12] Newman DB, Graham J, Diener E, Oishi S, Tay L (2018). Religion and well-being. Handbook of well-being.

[CR13] Newman DB, Schwarz N, Stone AA (2020). Global reports of well-being overestimate aggregated daily states of well-being. Journal of Positive Psychology.

[CR14] Nezlek JB, Matsumoto D, van de Vijer FJR (2010). Multilevel modeling and cross-cultural research. Cross-cultural research methods in psychology.

[CR15] Nezlek JB (2011). Multilevel modeling for social and personality psychology.

[CR16] Nezlek JB (2012). Diary methods for personality and social psychology.

[CR17] Nezlek, J. B. (2021a). Belief in God, well-being, and social capital: Distinguishing interpersonal and ideological prosociality. 10.17605/OSF.IO/RND6U

[CR100] Nezlek, J. B. (2021b). Ideological and Interpersonal Prosociality ESS8. osf.io/sncvy.

[CR18] Preston JL, Ritter RS, Ivan Hernandez J (2010). Principles of religious prosociality: A review and reformulation. Social and Personality Psychology Compass.

[CR19] Putnam RD (2000). Bowling alone: The collapse and revival of American Community.

[CR20] Raudenbush SW, Bryk AS (2002). Hierarchical linear models: Applications and data analysis methods.

[CR21] Raudenbush SW, Bryk AS, Cheong YF, Congdon RT, du Toit M (2011). HLM 7: Linear and nonlinear modeling.

[CR22] Schwartz, S. H. (2001). *European Social Survey Core Questionnaire Development –Chapter 7: A proposal for measuring value orientations across nations*.

[CR23] Stavrova O, Fetchenhauer D, Schlösser T (2013). Why are religious people happy? The effect of the social norm of religiosity across countries. Social Science Research.

[CR24] Stavrova O, Siegers P (2014). Religious prosociality and morality across cultures: How social enforcement of religion shapes the effects of personal religiosity on prosocial and moral attitudes and behaviors. Personality and Social Psychology Bulletin.

[CR25] Tay L, Li M, Myers D, Diener E, Kim-Prieto C (2014). Religiosity and subjective well-being: An international perspective. Religion and spirituality across cultures.

[CR26] UNESCO (2011). The International Standard Classification of Education (ISCED). Prospects.

